# Guide to Forensic Pathology Practice for Death Cases Related to Coronavirus Disease 2019 (COVID-19) (Trial Draft)[Author-notes FN0003]

**DOI:** 10.1080/20961790.2020.1744400

**Published:** 2020-04-13

**Authors:** Danmi Mao, Nan Zhou, Da Zheng, Jiacheng Yue, Qianhao Zhao, Bin Luo, Dawei Guan, Yiwu Zhou, Bingjie Hu, Jianding Cheng

**Affiliations:** Faculty of Forensic Medicine, Zhongshan School of Medicine, Sun Yat-Sen University, Guangzhou, China; Faculty of Forensic Medicine, Zhongshan School of Medicine, Sun Yat-Sen University, Guangzhou, China; Faculty of Forensic Medicine, Zhongshan School of Medicine, Sun Yat-Sen University, Guangzhou, China; Faculty of Forensic Medicine, Zhongshan School of Medicine, Sun Yat-Sen University, Guangzhou, China; Faculty of Forensic Medicine, Zhongshan School of Medicine, Sun Yat-Sen University, Guangzhou, China; Faculty of Forensic Medicine, Zhongshan School of Medicine, Sun Yat-Sen University, Guangzhou, China; School of Forensic Medicine, China Medical University, Shenyang, China; Department of Forensic Medicine, Tongji Medical College, Huazhong University of Science and Technology, Wuhan, China; Department of Pathology, Guangzhou Medical University, Guangzhou, China; Faculty of Forensic Medicine, Zhongshan School of Medicine, Sun Yat-Sen University, Guangzhou, China

**Keywords:** Forensic sciences, forensic pathology, autopsy, SARS-CoV-2, Coronavirus Disease 2019 (COVID-19), guide

## Abstract

Autopsy is of great significance for elucidating the pathological changes, pathogenesis and cause of death of Coronavirus Disease 2019 (COVID-19) and can provide a theoretical basis for scientific and accurate prevention and control of its outbreak. Based on related laws and regulations, such as the *Law of the People*’*s Republic of China on Prevention and Control of Infectious Diseases*, clinical manifestations and epidemiological characteristics of COVID-19 and guidelines on the prevention and control of this epidemic, combined with the practical work of forensic pathology examinations, we developed the *Guide to Forensic Pathology Practice for Death Cases Related to Coronavirus Disease 2019* (*COVID-19*) (*Trial Draft*). This guide describes the background investigation of the death cases, autopsy room requirements, personal prevention and protections, external examinations, autopsy practices and auxiliary examinations, and thus offers a reference for forensic and pathological examination institutions and staff.

## Introduction

Since December 2019, an outbreak of infection with the novel coronavirus (SARS-CoV-2) in China has progressed to many countries worldwide, raising concerns regarding Coronavirus Disease 2019 (COVID-19) endangering public health and life [1]. On 20 January 2020, COVID-19 was listed as a Category B infectious disease according to the *Law of the People*’*s Republic of China on Prevention and Control of Infectious Diseases* by the General Office of the National Health Commission of the People’s Republic of China, while the related prevention and control measures follow the standards of Category A infectious disease. On 30 January 2020, the World Health Organization declared the outbreak of COVID-19 a Public Health Emergency of International Concern.

With high infectivity, the incubation period of COVID-19 is approximately 14 days, and lasts for 24 days or more, in some patients [1–3]. One report [4] showed possible transmission between asymptomatic patients or between patients during the incubation period. A large amount of viruses may remain in deceased people infected with SARS-CoV-2, and the survival time of the viruses may be prolonged in refrigerated cadavers. During epidemic prevention and control, forensic pathologists may examine the body and tissues of the deceased infected with SARS-CoV-2 (including asymptomatic or suspicious infection). According to the *Law of the People’s Republic of China on Prevention and Control of Infectious Diseases* [5], *Regulations on biosafety management of pathogenic microorganism laboratories* [6], *Regulations on autopsy of patients with infectious diseases or suspected infectious diseases* [7], *Notice of guidelines for disposal of the remains of patients with pneumonia infected by novel coronavirus (trial version)* [8] and *Diagnosis and treatment of novel coronavirus pneumonia (trial version sixth)* [2], the guidelines in this article provide recommendations for biosafety and infection control practices during specimen collection and handling, as well as autopsy procedures.

## General rules for forensic pathology examination

1. Forensic institutions should follow the regulations of their national and local health departments when formulating work procedures and emergency plans for epidemic prevention and control.
2. To effectively confirm a SARS-CoV-2 infection or a suspected case, investigating the death process and medical history, and a case review are the first and most pivotal work in forensic autopsy regarding epidemic prevention.
3. Knowledge of COVID-19 should be updated timely, and the recommended practices and principles should be followed to avoid infection. Autopsy sites should have safety and protection protocols in place to guarantee personal and public safety.

## Contents of the forensic pathology examination

### Case investigation

Investigation involves collecting the deceased’s medical history and death process information obtained from clients (court or police), hospitals, family members, witnesses, funeral establishments, initial examiners and others. If necessary, clinicians are consulted to determine whether the deceased was a confirmed infection or a suspected case [2,9].

#### Suspected cases [2]

##### Epidemiological history

Fourteen days prior to symptom onset: (1) travel history or residence history in Wuhan or surrounding cities or communities reporting confirmed case(s); (2) close contacts of persons with COVID-19 (with positive results after nucleic acid testing); (3) exposure history to patients with fever or respiratory symptoms who came from Wuhan, surrounding cities or communities with confirmed case(s) and (4) evidence of clustering.

##### Clinical features

(1) fever and/or respiratory symptoms; (2) in early onset, the total number of white blood cells (WBC) was normal or decreased, or the lymphocyte count was decreased; and (3) imaging characteristics of COVID-19 as small patchy shadows and interstitial changes appeared early, especially in the lateral lung. Ground-glass opacities and infiltrates were seen subsequently in bilateral lungs. Lung consolidation might occur in severe cases.

A suspected COVID-19 patient must have one of the epidemiological history features and meet any two of the clinical features, or meet three clinical features and none of the epidemiological features.

#### Confirmed cases

A confirmed case must have one of the following two etiological evidences [2]: (1) nucleic acid-positive by real-time fluorescent quantitative polymerase chain reaction testing or (2) a high homology to SARS-CoV-2, as indicated by viral gene sequencing.

#### Latent cases

Asymptomatic infection and patients in the incubation period may become sources of infection; therefore, identifying latent cases is the most critical task for forensic pathologists.

People who do not meet the diagnostic criteria for suspected cases, but possess any of the following features, are recommended to undergo screening as possible latent cases: (1) meet some of the items listed for “suspected COVID-19” but have not reach the diagnostic thresholds for suspected cases yet; (2) have the following epidemiological history: travel history or residence history in Wuhan or surrounding cities or communities reporting confirmed cases, close contacts of persons with COVID-19 (with positive nucleic acid test results) and/or exposure history to patients coming from Wuhan or surrounding cities or communities with confirmed cases; (3) have the following clinical features: fever and/or respiratory symptoms, abnormal peripheral blood WBC count (with normal or reduced total WBC count or reduced lymphocyte count); and (4) imaging characteristics of SARS-CoV-2 infection.

### Categorising protection strategies

Before performing a postmortem examination, forensic examiners should consider the above criteria and determine whether a case is related to COVID-19, and if yes, determine the category for the case: *suspected, confirmed* or *latent*. After categorisation, protection and testing are performed according to the following principles:

#### Confirmed and suspected cases

Biosafety level 3 (BSL-3) protection should be provided during the scene investigation. The body, with clothes carefully removed, is gently moved to avoid blood and/or excrement contamination. Disinfectant containing 1 000 mg/L of chlorine should be used for on-site disinfection. Cadaver examination should be performed in the autopsy room with BSL-3 protection. The autopsy personnel’s clothing should meet the BSL-3 protection standards, strengthening the anti-splash protection for the face [6,10,11].

#### Latent cases

The site should be ventilated for at least 10 min before the crime scene investigation. Examiners should, with clothing following BSL-3 or BSL-2 protection standards, stand on the windward side and maintain a distance from the mouth and nose of the cadaver, and not press hard on the cadaver’s chest and abdomen. Cadavers can be examined in a conventional autopsy room, and examiners can dress according to BSL-3 protection standards. The disinfection is the same as that for confirmed cases.

#### Non-confirmed, non-suspected and non-latent cases

On-site and postmortem examination shall be performed under routine standards.

#### Unascertainable cases

During epidemic prevention, unknown corpses may be encountered in forensic examination, with no access to the deceased’s medical history or epidemiological history. With care, crime scene investigation and cadaver examination should be performed with protection levels as for suspected or latent cases, if necessary.

### Autopsy of confirmed and suspected cases

#### Cadaver examiner

A senior forensic pathologist must perform the examination. For safety reasons, at least two staff members should be present in the autopsy room at the same time. If possible, examiners should receive immunisation.

#### Autopsy site

An autopsy should be performed in an autopsy room with the following safe operating conditions [6]:

##### Requirements for an autopsy room

Cadaver examination should be performed in an autopsy room (with BSL-3 protection or equivalent conditions), with sufficient and sustained negative pressure, air and sewage discharge equipped with filtration or disinfection devices, and plane layout and ventilation according to general laboratory biosafety standards [10].

If an autopsy room with BSL-3 conditions is not available, cadavers can be examined in a disposable safety bag specially designed for postmortem examination of infectious diseases, which can completely isolate the infected body from examiners and surroundings. The examiner, from outside, performs the autopsy on the body in the transparent bag using safety sleeves and gloves.

In special circumstances, autopsy can be performed in an operating room with adequate negative pressure and air and sewage discharge equipped with filtration or disinfection devices.

##### Area division and safety operation procedures

The examination area, isolated from the surroundings, should be divided into three parts: clean area, semi-contaminated area and contaminated area. Using a single-channel closed-loop approach, examiners should move from the clean area to the semi-contaminated area and then to the contaminated area, and leave the contaminated area from another channel for the semi-contaminated area and then for the clean area. The semi-contaminated area, as a transition space, is the place for cleaning and disinfecting examiners, packaged materials and specimens.

##### Environmental disinfection

(1) Workplace: contaminated and semi-contaminated areas should be isolated before examination. During the examination, a 500 mg/L chlorine-containing disinfectant can be sprayed on the floors, walls and frequently contacted objects. After the examination, the autopsy room should be thoroughly sprayed and disinfected with a 1 000 mg/L chlorine-containing disinfectant. If the environmental surface is contaminated by secretions and excreta from the cadaver, the surface should be covered with hygroscopic materials and then disinfected with a 2 000 mg/L chlorine-containing disinfectant; (2) Apparatus and instrument: Before the examination, a 500 mg/L chlorine-containing disinfectant should be used to wipe tools and instruments, for disinfection. After the examination, a 1 000 mg/L chlorine-containing disinfectant is used for wiping and soaking for 30 min (or boiling in water for 30 min), and then clean water is used to rinse three times, followed by wiping clean for later use. Telephones, computer mouse and keyboards, cameras and other objects can be wiped with 75% ethanol solution disinfectants; (3) Air: ultraviolet ray disinfection lamps can be used for air irradiation disinfection for 1 h. Alternatively, ultra-low capacity sprays can be used, namely, 3% hydrogen peroxide, 5 000 mg/L peroxyacetic acid and 500 mg/L chlorine dioxide disinfectant (20–30 mL/m^3^) for 2 h. The doors and windows are closed during disinfecting, followed by full ventilation after disinfection.

#### Individual protection

Postmortem examination operators, including forensic pathologists, anatomical technicians, photographers, recording personnel and others should follow BSL-3 protection requirements; that is, wear a one-piece protective suit, grade ≥ N95 protective mask, goggles or protective eye mask, protective shoe covers and latex gloves (at least two layers). If liquid splash is possible, a respirator should be worn, and replaced immediately once contaminated.

#### Cadaver surface examination and autopsy

Before postmortem examination, according to relevant laws and regulations, the entrustment shall be ready, the deceased’s family should be informed and examination and emergency plans should be carefully prepared.

##### General protection

Before the examination, examiners should wear protective devices in the clean area, organise instruments used for autopsy in the semi-contaminated area, and then perform the autopsy in the contaminated area. After the examination, protective clothing and gloves must be sterilised for the first time in the autopsy room (contaminated area), and the surfaces of the protective clothing, mask, shoe covers and gloves should be thoroughly sterilised in the semi-contaminated area.

For objects, including cadavers, formalin-fixed specimens and frozen specimens that are taken from the autopsy room (contaminated area), the surfaces of the objects’ packaging containers should be thoroughly disinfected followed by packaging and sterilising the object again in the semi-contaminated area. Spray disinfection can be performed with 1 000 mg/L chlorine-containing disinfectants. The deceased’s clothing and items, such as gauze and towels used for the autopsy examination, should be disinfected and incinerated together with the cadaver.

##### Operation protection

Examiners should perform careful and gentle operations, with clear specialisation and close cooperation. Anatomical instruments should be used and placed properly to avoid puncturing gloves or skin with knives, scissors, sewing needles, puncture needles, syringes or the ends of fractured bones, as well as to avoid spattering blood, urine, gastrointestinal contents and bone powder. If gloves are torn, they should be disinfected and replaced immediately. If blood, body fluids, urine and/or faeces are splashed onto the examiner’s clothing or outside the autopsy table, strict disinfection should be performed. If the examiner’s skin is contaminated by cadaver pollutants, the pollutants should be removed immediately, and then disposable absorbent material with 0.5% iodine used to wipe the examiner’s skin for more than 3 min. Contaminated mucous membranes should be rinsed with large amounts of normal saline or 0.05% iodine to disinfect [12].

##### Autopsy and sample extraction

Prior to anatomical examination, a sample extraction plan can be made by consulting clinical pathologists, laboratory staff, physicians and virologists. To reduce the infection risks, samples should be taken directly after the body cavity is opened, and organ and tissue cuttings should be minimised [13].

Samples for etiological and electron microscopy testing and cryopreservation should be extracted first. Second, samples (secretions and tissue blocks) for etiological gene testing should be stored in Hanks’ solution. Third, tissues requiring freezing can be cut into blocks (∼1.5 cm in length, width and height) and put into a plastic bottle with a screw top before freezing. Fourth, tissues for electron microscopy examination can be cut into blocks (∼0.3 cm in length, width and height) and fixed in 3% glutaraldehyde. Finally, for specimens used for conventional paraffin embedding, entire diseased organs, tissues or tissue blocks with a length, width and height of 3–5 cm can be fixed in 4% paraformaldehyde solution for 48–72 h, and can then be examined, dehydrated, embedded and sectioned.

#### Formalin-fixed specimen preparation

##### Specimen collection

Fixed specimens can be retrieved in a well-ventilated laboratory in which surfaces and floors are sterilised with 2 000 mg/L chlorine-containing disinfectant and air is disinfected by ultraviolet radiation. After sampling, specimens are returned to the specimen bag with fixing solution and sealed. The material table is rinsed and then disinfected with 1 000 mg/L chlorine-containing disinfectant.

##### Tissue dehydration

Once dehydrated tissues are removed from the area, disinfect the surface of the dehydrator and surroundings with 1 000 mg/L chlorine-containing disinfectant for ∼30 min, and then wipe with clean water.

##### Tissue embedding

As for tissue dehydration, the embedding machine, surroundings and air are disinfected in two steps.

##### Tissue slicing

Paraffin blocks are sterilised by immersion in 75% ethanol solution and dried before slicing [12], and then sealed immediately and sterilised using 75% ethanol solution after slicing. The slicer is sprayed with 75% ethanol solution as well. Other tools such as forceps and scalpel and knife blades can be disinfected in an oven at 80 °C for 30 min.

Tissue staining: Instruments should be sprayed for disinfection with 75% ethanol solution or a 500 mg/L chlorine-containing disinfectant both before and after use.

#### Effluent and waste disposal

Generation of effluent and waste should be avoided as much as possible. Infectious effluent, the waste water produced during examination, should be treated by chemical or physical disinfection and discharged after complete inactivation [12]. Solid wastes, including consumables, personal protective equipment and any remaining fixed specimens should be collected separately for processing. Consumable and personal protective equipment should be sterilised using high-pressure steam or fumigated using ethylene oxide in a timely manner.

#### Auxiliary examination

In addition to pathomorphological observation of routine paraffin sections, hematoxylin-eosin staining, special staining, immunohistochemical staining and immunofluorescence staining, virus isolation and gene sequencing of secretions and tissue blocks, *in situ* detection of viral RNA or viral protein antigens in tissue sections, ultrastructural examination of tissue sections and detection of virus particles can be performed, if necessary.

#### Results documentation

Forensic autopsy is currently the main way to identify and accumulate systematic pathological information for death cases. Examination agencies should fully record and maintain the basic information for COVID-19 patients (name, age, sex, place of origin, place of residence, place of onset and travel history), information from the anatomical examination and epidemiological and clinical data. Feedback on pathology examination reports or the identification opinion of the cause of death should be timely sent to related medical institutes, disease prevention control agencies and health administration departments [7,11].

#### Others

During the epidemic outbreak, all operators should undergo medical observation after autopsy, and those with fever and respiratory symptoms should be isolated for observation or medical assistance. Operators involved in the autopsy of confirmed or suspected cases should receive temperature monitoring and medical observation in isolation for 14 days.

Viruses have certain survivability in cadavers and *in vitro* (Appendix A). It is not uncommon for examiners to be infected secondary to inadequate protection, careless operation or unknown causes (Appendix B). Therefore, during the epidemic prevention and control period, personal protection of examiners and disinfection of dissecting rooms, surroundings and instruments should be taken seriously.

If a glove is torn and skin is damaged during autopsy, the operator should stop dissecting immediately, rinse the wound with large volumes of saline or a 0.05% iodophor for disinfection, and be isolated for treatment and observation.

If new confirmed or suspected cases of COVID-19 are found during examination, operators should report to the local disease prevention and control agencies or medical institutes in a timely manner.

## Appendix A: Viral survival times in cadavers and *in vitro*

According to studies of other coronaviruses in the same genus, it is inferred that the SARS-CoV-2 virus has strong persistence in the external environment, especially at low temperatures.

According to a study on the resistance of severe acute respiratory syndrome corona virus [14], the virus can survive for 2 days in hospitals or domestic sewage and dechlorinated tap water, for 3 days in faeces, 14 days in normal saline and 17 days in urine at 20°C away from light. However, at 4°C, the virus can survive longer than 14 days in the abovementioned water settings, and longer than 17 days in faeces. In a case report of a patient dying with Middle Eastern respiratory syndrome corona virus [15], the virus was detected in nasal secretions even 3 days after death. Kampf et al. [16] summarised the persistence of coronavirus on different inanimate surfaces and at different temperatures.

## Appendix B: Cases of pathogen transmission and infection during autopsy

Studies reported that infections occurred secondary to attending, observing or learning autopsies performed on people who died from infectious diseases (Table A1). Nolte et al. [17] reported that operators are highly likely to be infected with pathogens during the autopsy of victims of infectious diseases when gloves and skin are punctured by sharp objects such as broken bones, anatomical instruments and needles. Therefore, prevention and control measures should be considered seriously in postmortem examination in infectious disease deaths.

**Table A1:** Cases of infection of examiners during autopsy.

Microbe	Cause of infection	Incubation period	References
Mycobacterium tuberculosis	Airborne transmission	Unknown	[18–23]
Streptococcus pyogenes	Contact transmission (finger scratched)	16 d	[24,25]
Ebola virus	Contact transmission (finger scratched)	12 d	[26]
Blastomyces	Contact transmission (finger scratched)	3–5 w	[27]
Prion	Unknown	> 10 y	[28]
Hepatitis virus	Contact transmission (blood)	Unknown	[20,29–31]
HIV	Contact transmission (finger scratched)	Unknown	[31,32]
